# CRISPR-mediated optogene expression from a cell-specific endogenous promoter in retinal ON-bipolar cells to restore vision

**DOI:** 10.3389/fddev.2023.934394

**Published:** 2023-03-27

**Authors:** A. Maddalena, S. Kleinlogel

**Affiliations:** ^1^ Institute of Physiology, University of Bern, Bern, Switzerland; ^2^ Department of Biomedical Research (DBMR), University of Bern, Bern, Switzerland; ^3^ Roche Pharma Research and Early Development, Neuroscience and Rare Diseases, Roche Innovation Center Basel, F. Hoffmann-La Roche-Ltd, Basel, Switzerland

**Keywords:** optogenetics, CRISPR/Cas9-mediated genome editing, vision restoration, gene therapy, exo-AAV, Opto-mGluR6, HITI, MITI

## Abstract

Retinitis pigmentosa, an inherited form of retinal degeneration, is characterized by a progressive loss of rods and subsequent degeneration of cones, leading to blindness. However, the remaining neural portion of the retina (bipolar and ganglion cells) remains anatomically and functionally intact for an extended time. A possible treatment to restore the light sensitivity of the retina consists of rendering the remaining retinal cells photosensitive using optogenetic tools like, for example, Opto-mGluR6, a light-sensitive mGluR6 receptor. We have previously demonstrated that AAV vector-mediated expression of Opto-mGluR6 in ON-bipolar cells restores visual function in otherwise blind mice. However, classical gene supplementation therapy still suffers from high off-target expression rates and uncontrollable target gene expression levels that may lead to either cytotoxicity or lack of functional restoration. To address these issues and achieve cell-specific and endogenously controlled Opto-mGluR6 expression, we employed the CRISPR/Cas technology—in particular, homology-independent targeted integration (HITI) and microhomology-dependent targeted integration (MITI)—to knock-in the Opto-mGluR6 gene behind the ON-bipolar cell-specific GRM6 promoter. We compared four Cas systems *in vitro* and show that SpCas9 for HITI and LbCpf1 for MITI are well suited to promoting knock-in. As AAV2-mediated ON-bipolar cell transduction resulted in inefficiency, we evaluated Exo-AAVs as delivery vehicles and found Exo-AAV1 efficient for targeting ON-bipolar cells. We demonstrate that intravitreal injection of Exo-AAV1 carrying vectors that promote MITI significantly improved visual acuity in otherwise blind *rd1* mice. We conclude by confirming and providing a qualitative evaluation of the MITI-mediated knock-in in the correct genomic locus.

## 1 Introduction

Retinitis pigmentosa (RP) is an inherited form of retinal degeneration that affects, on average, 1 in 3,500 people. RP is a very heterogeneous disease; mutations in more than 80 genes, especially found in retinal pigment epithelial cells and rod photoreceptors (PRs), have been linked to it (Daiger et al., 2013). The disease is characterized by a progressive loss of rods and subsequent degeneration of cones, ultimately leading to blindness. In the early stages of this disease, PR-preserving gene-replacement therapies are employed (Bainbridge et al., 2008; Testa et al., 2013). Retinal gene therapy typically employs adeno-associated virus (AAV), the only viral vector able to efficiently transduce different retinal cell types (Day et al., 2014). In 2017, the first AAV-based retinal gene therapy targeting the *RPE65* gene in retinal epithelial cells (Luxturna) received FDA and EMA approval (Testa et al., 2013). Due to the heterogeneity of RP, a comprehensive gene therapy is difficult to implement. In addition, patients often seek help only in the advanced stages of degeneration, beyond the stage where PRs can be saved. The remaining neural portion of the retina (bipolar and ganglion cells), however, remains anatomically and functionally intact beyond the death of PRs (Strettoi and Pignatelli, 2000; Chang et al., 2002). This has led to the development of various therapeutic strategies, such as implanting stem cells to replace lost PRs (Bellapianta et al., 2022; Li et al., 2013), retinal prostheses electrically activating the remaining neural retina (Weiland and Humayun, 2014), or the use of synthetic photo-switchable ligands (Polosukhina et al., 2012) or optogenetic proteins that turn bipolar or ganglion cells into light-sensitive “replacement photoreceptors” (Bi et al., 2006; Lagali et al., 2008; Cehajic-Kapetanovic et al., 2015; van Wyk et al., 2015; Sengupta et al., 2016; Berry et al., 2019). Two optogenetic gene therapies aiming to express channelrhodopsin-2 (ChR2) in retinal ganglion cells—the output neurons of the retina—have recently entered clinical trial to restore vision in patients suffering from advanced RP (NCT02556736 and NCT03326336). Data from the first patient confirmed the safety of the approach and the restoration of basic visual function (Sahel et al., 2021).

Recent refinements of the optogenetic vision restoration strategy have focused on enabling the expression of the optogenetic proteins within the first-order retinal interneurons—the retinal ON-bipolar cells (ON-BPCs)—to diversify restored retinal signaling and thus enhance the quality of restored vision (Ivanova et al., 2010; Cronin et al., 2014; Hulliger et al., 2020). In addition, designer optogenetic proteins are being developed that couple the light signal with the endogenous G-protein signaling pathway of ON-BPC (Cehajic-Kapetanovic et al., 2015; van Wyk et al., 2015; Berry et al., 2019). Opto-mGluR6 is a chimeric G-protein-coupled-receptor (GPCR) that consists of the intracellular domains of the ON-BPC-specific metabotropic glutamate receptor, mGluR6, and the light-sensing domains of melanopsin, a blue-light-sensitive retinal photopigment (Sekharan et al., 2012). Opto-mGluR6, a chimera of proteins of human origin, is about 500 times more light sensitive than ChR2 and naturally triggers endogenous TRPM1 signaling in ON-BPCs (van Wyk et al., 2015). We have previously demonstrated that the intravitreal injection of AAVs carrying the Opto-mGluR6 optogene under the control of the ON-BPC-specific GRM6 promoter (Hulliger et al., 2020) can restore visual function in otherwise blind retinal degeneration (*rd1*) mice (van Wyk et al., 2015; Kralik and Kleinlogel, 2021).

Despite the many advantages of the Opto-mGluR6 ON-BPC targeted gene therapy, there remain points for improvement. (1) A gene therapy is never 100% cell-specific, and off-target Opto-mGluR6 expression may lead to corrupt signaling within the retinal network, ultimately diminishing the quality of restored vision. (2) AAV gene therapies require the use of short enhancer/promoter sequences due to the small packaging capacity of the AAV capsid (approx. 4.8 kb) (Hermonat et al., 1997). Such short promoters will not contain all regulatory elements that lead to the fully endogenous regulation of gene expression—either reducing functional efficacy or inducing cytotoxicity due to protein over-expression. (3) Expression of the native mGluR6 receptor, which signals in the dark—as opposed to Opto-mGluR6, which is activated by light—may lead to signal confusion within the ON-BPCs in patients with incomplete or patchy PR degeneration.

In order to overcome these issues, we sought to place the coding sequence of Opto-mGluR6 under the control of the endogenous, ON-BPC-specific GRM6 promoter using CRISPR/Cas genome editing (Doudna and Charpentier, 2014). We compared two CRISPR/Cas-based systems: the homology-independent targeted integration (HITI) system (Suzuki et al., 2016; Suzuki and Izpisua Belmonte, 2018) and the microhomology-independent targeted integration (MITI) system (Li et al., 2019). Both strategies exploit the non-homologous end-joining (NHEJ) DNA repair mechanism, allowing exogenous DNA, such as Opto-mGluR6, to be inserted into the genomic DNA of the target cell.

**FIGURE 1:**
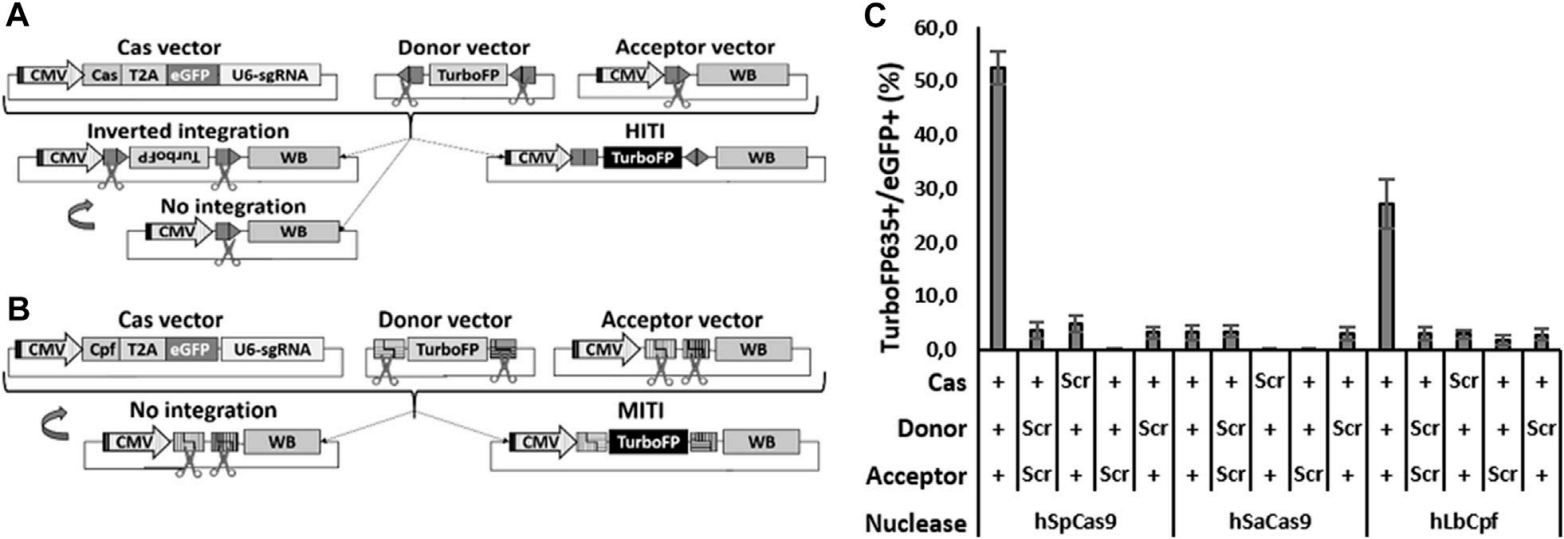
Efficient HITI and MITI with human sgRNAs in N2a cells. **(A, B)** Schematic representation of the vectors for *in vitro* HITI **(A)** and MITI **(B)**. The TurboFP635 sequence is indicated in light gray when the gene is not expressed, while it is indicated in black when the protein is expressed. CMV: cytomegalovirus ubiquitous promoter; T2A, T2A peptide; U6-sgRNA, sgRNA expression cassette; WB, WPRE-BGHpA. **(C)** Cytofluorimetric quantification of HITI and MITI. Scr: scramble plasmid; RFP: TurboFP635 expressing vector. Shown are mean ± sem, *n* = 3 experimental replicates.

The HITI strategy is based on the use of the Cas9 protein which, upon cutting the DNA between the seeding region and the protospacer adjacent motive (PAM), releases blunt ends; a selected single-guide (sg) RNA target sequence is then placed in a reverse orientation at the two extremities of the donor DNA template to be inserted. Cas9 cuts the target sites both in the genome and in the donor DNA; in case of correct recombination, the target sites will be destroyed, while, in case of inverted or no integration, the Cas9 target sites will be reformed, favoring a second round of cutting (Figure 1A). The HITI system has already been successfully used in the retina of Royal College of Surgeons (RCS) rats, a well-established rat model of RP resulting from a homozygous 1.9-kb deletion within intron 1/exon 2 of the Mertk gene. Using HITI, the authors knocked-in the missing part of the Mertk exon 2, which resulted in a partial morphological rescue of the photoreceptors and a functional rescue of vision (Suzuki et al., 2016). In a similar study, Tornabene and colleagues partially restored the retinal phenotype of P23H mice—a widely used mouse model for RP (Tornabene et al., 2022).

On the other hand, the MITI strategy, based on the Cpf1 (or Cas12) nuclease, releases sticky ends upon DNA cleavage. In this case, two distinct nuclease genomic target sites must be identified, the sequences of which are modified in the donor vector in order to release the DNA template flanked by compatible sticky ends. As with HITI, only a directional KI will destroy the target sites, while a lack of recombination would reconstitute the target sites, favoring another round of cutting. As opposed to HITI, an inverted integration should not be possible with MITI (Figure 1B).

In this study, we aimed to place Opto-mGluR6 expression under the control of the endogenous GRM6 promoter, which is only active in retinal ON-BPCs (Siegert et al., 2012). We tested four different Cas proteins for their *in vitro* efficacies for targeted genomic Opto-mGluR6 insertion, promoting HITI—the widely used *Staphylococcus pyrogens* (Sp) Cas9 and the smaller *S. aureus* (Sa) Cas9 (Ran et al., 2015)—or MITI—the *Lachnospiraceae bacterium* LbCpf1 (Zetsche et al., 2015; Ye et al., 2016; Li et al., 2019) and the *Acidaminococcus sp*. BV3L6 AsCpf1 (Zetsche et al., 2017). *In vitro,* SpCas9 outperformed SaCas9, and LbCpf1 showed higher efficacy than AsCpf1. Both SpCas9 and LbCpf1 were tested to mediate Opto-mGluR6 KI with ON-BPCs *in vivo*. To accomplish high-efficacy dual AAV transduction of ON-BPCs after intravitreal injection into the eyes of retinal degeneration (*rd1)* mice, we exploited Exo-AAVs: small vesicles filled with AAVs released by AAV-producing cells during AAV production. Using our therapy, we found a significant improvement of visual acuity in *rd1* mice injected with Exo-AAV1 particles that enabled CRISPR-mediated Opto-mGluR6 KI. The functional restoration was supported by next-generation sequencing (NGS) analysis showing seamless Opto-mGluR6 insertion behind the Grm6 promoter in ON-BPCs.

## 2 Materials and methods

### 2.1 Plasmid and AAV preparation

For sgRNA selection, we used bicistronic plasmids, with one cassette bearing the nuclease CDS fused *via* a T2A sequence to eGFP and another cassette with an U6 promoter and a specific type IIS cloning site for the sgRNA sequence insertion. Specifically, we used pSpCas9(BB)-2A-GFP (pX458), a gift from Feng Zhang (Addgene plasmid # 48138) (Ran et al., 2013); pX601-GFP (saCas9), a gift from Yuet Wai Kan (Addgene plasmid # 84040) (Ye et al., 2016); pY095 (LbCpf1), a gift from Feng Zhang (Addgene plasmid # 84744) (Zetsche et al., 2017); and pY094 (AsCpf1), a gift from Feng Zhang (Addgene plasmid # 84743) (Zetsche et al., 2017). Each sgRNA was ordered from Microsynth AG as a couple of single-stranded DNA oligonucleotides. For the annealing, each oligo DNA was resuspended at a 100 mM concentration. Oligo pairs were mixed at a final concentration of 10 mM with annealing buffer (10X: 20 mM Tris, pH 7.8, 100 mM NaCl, and 0.2 mM EDTA) in a total volume of 20 µL, heated for 5 min at 95°C, and cooled by 1°C per 12 s until they reached room temperature. The solution was then spun down and diluted to 1:200. Finally, 2 μL was used for the ligation with 100 ng of the respective plasmid cut with the dedicated type IIS restriction enzyme—the pX458 was cut with BbsI, pX601 with BsaI, and both pY094 and pY095 with BsmBI.

For AAV vector generation, we used an AAV backbone with AAV2 ITRs while, for non-AAV vectors, we used the pIRES backbone. For details of vector composition, see (van Wyk et al., 2017; Hulliger et al., 2020; Kralik and Kleinlogel, 2021). The miniCMV promoter was derived from plasmid pX551-miniCMV-SpCas9 (Addgene plasmid # 107031). The plasmids encoding 5′ CDH23, the sequences of the splicing donor and acceptor signals (CDH-AAV 1 and 2) (Maddalena et al., 2018), and the spacer sequence were derived from a non-coding region of the adenovirus genome [ED-AAV1 (Maddalena et al., 2018)]; these were a kind gift from Alberto Auricchio (TIGEM, Naples, Italy). The PCR amplified sequence of ED-AAV1 is as follows:

ggc​cca​cgg​gcg​gcg​gcc​tgg​gcg​aag​ata​ttt​ctg​gga​tca​cta​acg​tca​tag​ttg​tgt​tcc​agg​atg​aga​tcg​tca​tag​gcC​att​ttt​aca​aag​cgc​ggg​cgg​agg​gtg​cca​gac​tgc​ggt​ata​atg​gtt​cca​tcc​ggc​cca​ggg​gcg​tag​tta​ccc​tca​cag​att​tgc​att​tcc​cac.

AAVs were packaged as described previously (Kralik et al., 2022).

### 2.2 Cell culture and transfection

HEK293 cells (ATCC) were cultured in complete media consisting of Dulbecco’s modified Eagle medium (DMEM; Sigma, D5671), supplemented with 10% fetal calf serum (FCS; Seraglob, S70500), 1X glutamine (Seraglob, K8701), and 1% penicillin/streptomycin (Sigma, P0781), and were grown at 37°C and 5% CO_2_. Plasmid transfection was performed using the calcium phosphate precipitation method; briefly, the DNA was resuspended in 25 mM calcium solution and an equal amount of 2X HEPES buffered saline (HBS) was added to the mix. The mix was added to the cells 3 to 5 min later and incubated for 4–6 h. The media were then exchanged with DMEM with 2% FBS, supplemented with 1X glutamine and 1% penicillin/streptomycin. When 6-well plates were used, 1 × 10 ^6^ cells/well were seeded in 2 mL the day before transfection, and 1–2 µg of DNA and 150 µL of both calcium solution and 2X HBS solution were used. When 24-well plates were used, 2.5 × 10^5^ cells were seeded in 500 µL the day before transfection, and 100 ng of DNA (when dual transfections were performed, we used 100 ng for each plasmid) and 30 µL of both calcium solution and 2X HBS solution were used.

Murine N2a cells were a kind gift from Prof. Stephan Rohr (Institute of Physiology, Bern, CH). These were grown in Eagle’s minimum essential media (EMEM; Sigma, M5775) supplemented by 10% fetal calf serum (FCS; Seraglob, S70500) and 1% penicillin/streptomycin (Sigma, P0781), and grown at 37°C and 5% CO_2_. Plasmid transfection was performed using the TransIT^®^LT 1 reagent (Mirus Bio, MIR2300). Briefly, the DNA and the transfection reagent were mixed in Opti-MEM medium (Sigma, 31985062), vortexed, and incubated at RT for 20–30 min before addition to the cells. When 6-well plates were used, 3 × 10^5^ cells/well were seeded in 2 mL the day before transfection, and 3 µg of DNA and 6 μL of TransIT®LT 1 reagent were mixed in 100 µL of Opti-MEM. When 24-well/plates were used, 1.5 × 10^5^ cells/well were seeded in 500 μL the day before transfection, and 400 ng of DNA (when multiple transfections were performed, we used 400 ng for each plasmid) and 3 μL of TransIT^®^LT 1 reagent were mixed in 50 µL of Opti-MEM.

### 2.3 sgRNA selection and INDELs (insertions/deletions) analysis

The sgRNA targets were identified within exon 1 and intron 2 of both human (NG_008105.1 from bp 1 to 23797) and mouse (NC_000077.7 from bp 50741195 to 50757035) *GRM6* genes using the online Benchling tool (www.benchling.com). For the selection, we took care to pick non-overlapping sequences with a high on-target value and avoid cutting next to a splicing site. For LbCpf1 and AsCpf1, we used the same criteria, but we selected targets with both positive and negative orientations. For SaCas9 sgRNAs, we considered the design proposed by Ran and colleagues to use a seeding sequence of 23 bp (Ran et al., 2015), while for the Cpf1 sgRNAs we used the design proposed by Bin Moon and colleagues with a 23-bp seeding sequence and a modified poliT (Bin Moon et al., 2018).

sgRNA plasmids were used to transfect HEK293 cells (for human targets) or N2a cells (for murine targets) in 6-well plates. Three days post-transfection, the cells were washed with PBS, detached using Trypsin/EDTA, harvested, spun down at 800 g for 4 min, washed with PBS, and resuspended with 400 μL PBS complemented with 2% FCS. For INDEL analysis, at least 50.000 eGFP-positive cells were isolated by fluorescence-activated cell sorting (FACS) using a BD FACSAria™ III Cell Sorter (BD bioscience), and the genomic DNA was isolated using the ReliaPrep gDNA Tissue Miniprep System (Promega, A2052). The area around the predicted sgRNA target sites were PCR amplified in order to obtain an 800–1,000-bp product, with one end 100–300 bp from the respective target site. Primers were ordered from Microsynth AG, and the PCR was performed using the Herculase II PCR kit (Agilent, 600675). The PCR product was isolated using the gel and PCR cleanup kit (Macherey-Nagel, 740609) and sent for sequencing to Microsynth using the primer closer to the target site as a sequencing primer. Results from control samples and treated samples were compared using the online tool Tracking of Indels by DEcomposition (TIDE: https://tide.nki.nl/), which gives an estimation of the amount of INDELs produced and, consequentially, the efficiency of the sgRNA.

### 2.4 AAV and Exo-AAV production

Viral vectors were produced in AAV-293 cells (ATCC CRL-1573) by triple plasmid co-transfection. We co-transfected the different AAV vectors, the AAV-helper plasmid encoding Rep2 and Cap2 (7m8) (Addgene plasmid # 64839) (Dalkara et al., 2013), and the pXX680 plasmid-harboring helper adenoviral genes (kindly provided by H. Büning) using the calcium phosphate precipitation method. Empty virions were removed by density purification over an iodixanol gradient (Axis-Shield, Oslo) and the 40% iodixanol fraction subsequently buffer-exchanged by Amicon filtration (Millipore). The AAV fraction was titrated for DNase-resistant vector genomes by real-time PCR relative to a standard vector.

For Exo-AAV production, 1 × 10^7^ HEK293T cells/plate were seeded in 1–4 15-cm plates in 30 mL of DMEM 10% complete media and were transfected using the calcium phosphate precipitation method. Briefly, for each plate, 26 μg of pXX680, 12 μg of Rep and Cap plasmids, 9 μg of aap2 plasmid, and 12 μg of AAV plasmid were added to 1 mL of 25 mM calcium solution. The Rep2 Cap1 plasmid was a gift from James M. Wilson (Addgene plasmid # 112862). A measure of 1 ml of 2X HBS solution was slowly added under constant vortexing, and the final solution was left to rest for 5 min. The mix was then added dropwise to the plates, and the media was exchanged 6 h later with DMEM exosome-free 2% FCS complete media. Exosome-free FCS was obtained by centrifuging FCS for 1 h at 100.000 g. Three days after transfection, the Exo-AAV-containing supernatant was collected and centrifuged for 10 min at 300 g and 10 min at 2,000 g. The different preparations containing virions with different transgenes were then combined within the two subsequent centrifugation steps for 1 h at 20.000 g and 1 h at 100.000 g. The supernatant was discarded, and the tubes were carefully air-dried. The final pellet was then resuspended in 50–200 µL PBS and stored at −80°C in 20–30 µL aliquots until use. Titration was performed by real-time qPCR relative to a standard vector.

### 2.5 Animal experiments

Animal experiments and procedures were in accordance with the Swiss Federal Animal Protection Act and approved by the animal research committee of Bern (approval number BE44-12). C57BL/6, C3H/HeOuJ (*rd1*), and FVB/N_OptomGluR6/rd1 mice were bred in-house and kept on a 12-h light/dark cycle.

#### 2.5.1 Vector injection

Anesthesia was induced by 5% and maintained with 2% isoflurane in 100% O_2_. The mice were placed under a stereotaxic microscope, and the pupils of their eyes were dilated with a drop of 10 mg/mL atropine sulfate (Théa Pharma). We then punctured the dorsal sclera approximately 1 mm from the corneal limbus using an insulin needle. This was removed, and a 33-G blunt needle was maneuvered through the pre-made hole to the back of the eye (RPE injection kit from World Precision Instruments). We then injected 1–2 μL of the AAV vector or Exo-AAV solution intravitreally and waited 2 min before retracting the injection needle from the eye. Following surgery, an antibiotic eye lotion (Isathal from Dechra Veterinary Products) was applied to the eyes to prevent infection and drying of the cornea. The animals were left for a few minutes to awaken and then placed back in their cage.

#### 2.5.2 Optomotor reflex (OMR) measurements

The animals were brought to the experimental room the night before testing for adaptation. We employed a Striatech’s LCD monitor-based virtual automated optomotor system to assess the spatial frequency thresholds of specific optomotor behavior in awake, unrestrained mice sitting on an elevated platform (9-cm diameter, 10-cm height). The brightness of the screens was adjusted to 5 × 10^13^ photons cm^−2^ s^−1^. The head movements of mice in response to horizontally moving (clockwise and counterclockwise) sinusoidal gratings were tracked from above by an infrared-sensitive digital camera and scored using OptoDrum software (Striatech, v.1.5.2). The rotation speed was kept constant at 12°/s, which was shown to elicit an optimum response under photopic conditions (Umino et al., 2008). Visual acuity was examined with a stair-step protocol of increasing spatial frequencies (100% contrast, speed 12°/sec), and threshold was defined as the highest grating frequency that can evoke animal head tracking (Prusky et al., 2004; Benkner et al., 2013). Right and left eyes were measured separately by changing the rotation direction (clockwise for the left eye and counterclockwise for the right). The animals were tested six times within 2 weeks, and the six measurements were averaged.

#### 2.5.3 Immunohistochemistry

At the end of the experiment, the mice were euthanized, and their retinas were extracted for subsequent immunohistochemistry. The retinas were dissected and fixed in 4% paraformaldehyde in 0.1 M phosphate buffer (pH 7.4) for 45 min. For frozen sections, the retinas were cryoprotected in graded sucrose solutions before freezing in OCT medium (Sakura Finetek). Then, 14-μm-thick cryosections were mounted on SuperFrost slides (Menzel). Antibodies were diluted in a blocking solution containing 0.3% Triton-X, 1% bovine serum albumin (BSA, Sigma, A2153), and 5% of normal goat serum (NGS, Sigma, G9023) in TBS (150 mM NaCl, 50mMTris-HCl pH7.5). Cryosections were incubated for 1 h in blocking solution, overnight at 4°C in primary antibody and 2 h in secondary antibody at room temperature. We used the following antibodies: rabbit anti-GFP 1:500 (Invitrogen, A11122); goat anti-Gαo 1:/750 (Millipore, MAB3073); rabbit anti-tRFP 1:500 (Evrogen, AB234); chicken anti-GFP 1:500 (Abcam, ab13970); rabbit anti-GFAP 1:500 (DAKO, Z0334); rabbit anti-Iba1 1:500 (WAKO, 19-19741); rat anti-CD11b 1:100 (BioLegend, 101202); rabbit anti-RBPMS 1:500 (Merck Millipore, ABN1362); donkey anti-chicken 1:400 (Abcam, ab150169); goat anti-rabbit Alexa 488 1:400 (Invitrogen, A11008); goat anti-mouse CY3 1:400 (Invitrogen, A10521); and goat anti-rabbit Alexa 594 1:400 (Invitrogen, A11037). Nuclei were stained with 10 μg/mL DAPI (Roche). Sections were analyzed either under a Zeiss inverted microscope, equipped with Axiocam 712 mono-camera and ZEISS-Blue software, or under an ANA Zeiss LSM 880 confocal microscope (equipment supported by the Microscopy Imaging Center (MIC), University of Bern, Switzerland). Images were then processed and analyzed using ImageJ (Rasband WS, United States National Institutes of Health, Bethesda, Maryland, United States). For cell counting, counts from three to six 40× images (212 μm × 212 μm) stained against eGFP and Gαo were averaged.

#### 2.5.4 Genomic analysis

We used C3H/HeOuJ_Opto-mGluR6 mice, a transgenic mouse line expressing TurboFP635 exclusively in ON-BPC (van Wyk et al., 2015). Four 5-week-old mice were intravitreally injected, as previously described, and were sacrificed 5 weeks later. Their eyes were enucleated and the retinas dissociated in L-15 Leibovitz’s medium (Sigma, L5520) supplemented with 5% glutamine and 15% FCS using the Papain dissociation system (Worthington, LK003150). TurboFP635-positive and -negative cells were sorted using a BD FACSAria™ III Cell Sorter. Genomic DNA was extracted using the AllPrep kit (QIAGEN, Cat. 80204). Primers were ordered from Microsynth AG, and the PCR was performed using the Herculase II PCR kit (Agilent, 600675). In the first PCR, we used the forward primer TGT​CCG​GCC​AGA​ATC​CCG​AA and reverse primer CCA​AAC​GCC​CAG​CAG​GAC​AA. In the second (nested) PCR, we used the forward primer TGA​AGA​AGG​AGC​AGG​GAG​TG and reverse primer AAA​TGC​CAA​AGA​GAG​CTC​CA. Both PCRs underwent a first denaturation step of 3 min at 98°C, followed by 45 cycles of 15 s denaturation at 98°C, 20 s of annealing at 60°C, and 45 or 30 s, respectively, at 72°C for elongation, followed by 3 min at 72°C for final elongation. Both PCRs were run in 2% agarose gel and the products purified using a PCR and gel purification kit (NucleoSpin, 740609). The quantity, purity, and length of the amplified DNA samples were assessed using a Thermo Fisher Scientific Qubit 4.0 Fluorometer with the Qubit dsDNA HS Assay Kit (Thermo Fisher Scientific, Q32854) and an Agilent Fragment Analyzer (Agilent) with a HS NGS Fragment Kit (Agilent, DNF-474), respectively. Sequencing libraries were made using an Illumina Nextera XT Library Preparation Kit (Illumina, FC-131-1096) in combination with Nextera XT Index Kit v2 Set A (Illumina, FC-131-2001) according to the Illumina Nextera XT Reference Guide notes for large amplicons (Illumina, 15031942v01). The large amplicon libraries were assessed for quantity and quality, as described previously, using fluorometry and capillary electrophoresis. Pooled DNA libraries were sequenced paired-end on a MiSeq Reagent Nano Kit v2 (500 cycles; Illumina, MS-103-1003) on an Illumina MiSeq instrument. The quality of the sequencing run was evaluated using the Illumina Sequencing Analysis Viewer (Illumina version 2.4.7), and all base call files were demultiplexed and converted into FASTQ files using Illumina bcl2fastq conversion software v2.20. All steps from amplicon QC to sequencing data generation were performed on the Next-Generation Sequencing Platform, University of Bern, Switzerland. The data were then analyzed by the Interfaculty Bioinformatics Unit of the University of Bern, and a region of ±50 bp around the insertion point was considered for the analysis.

#### 2.5.5 Statistical analysis

**FIGURE 2:**
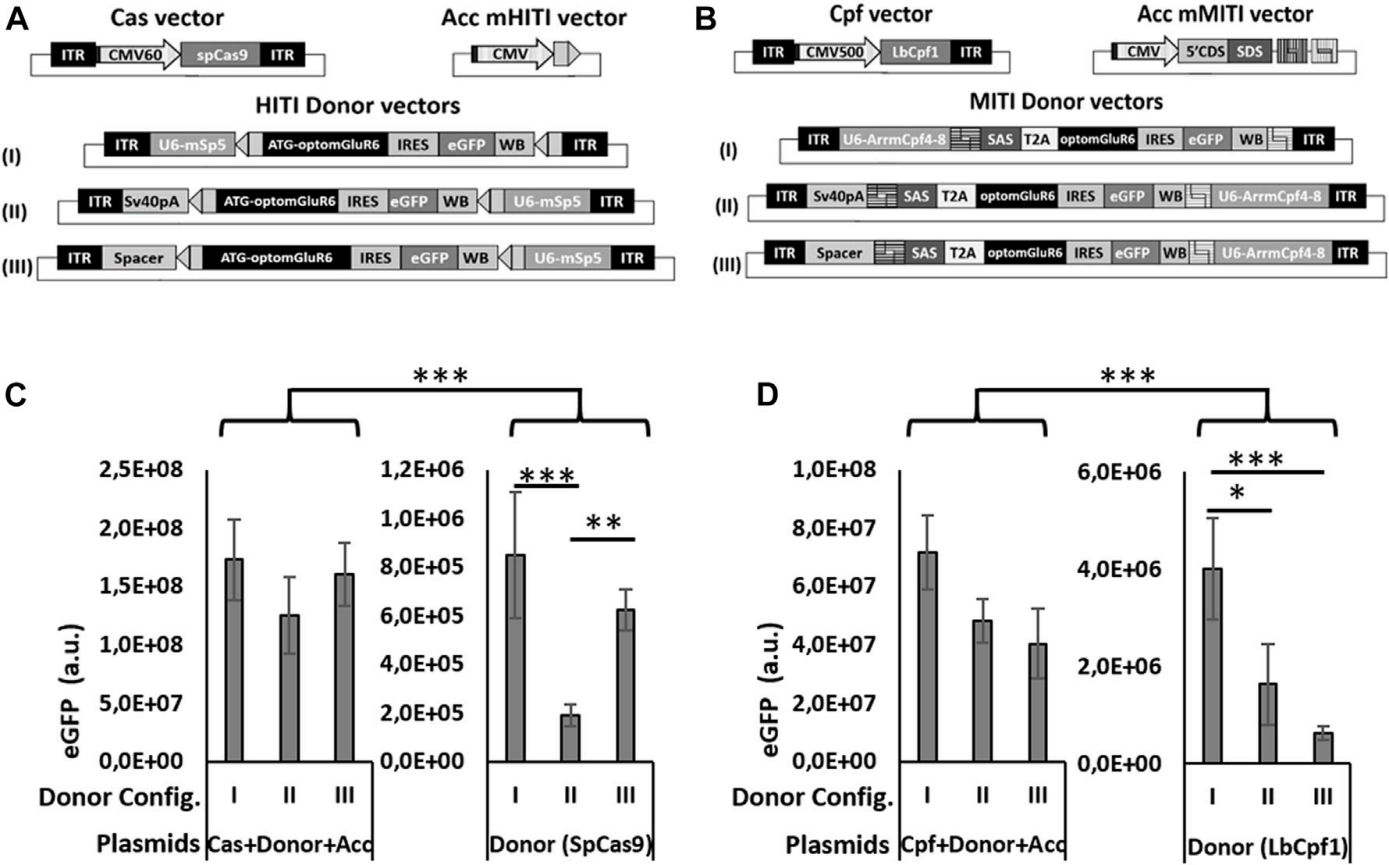
Different configurations of donor vectors show different efficiencies in HITI/MITI and different background expression. **(A, B)** Schematic representation of the set of vectors for HITI **(A)** and MITI **(B)** with murine sgRNAs and **(C, D)** quantification of eGFP expression (number of eGFP-positive cells x mean eGFP intensity) measured by cytofluorimetric analysis in HEK293 cells transfected with the A & B indicated sets of plasmids. *p < 0.05; **p < 0.01. ***p < 0.001, a.u. = arbitrary units, *n* = 5 experimental replicates.

Statistical *p*-values ≤ 0.05 were considered significant. We performed two-way ANOVA with a Bonferroni *post hoc* test (Figure 2). Behavioral data were analyzed with one-way ANOVA for multiple comparisons with *post hoc* analysis using Tukey’s honestly significant difference (HSD) of means in GraphPad Prism v7. Assumptions of normality were not rejected by the Shapiro–Wilk normality test, and homogeneity of variance was tested with Levene’s test. Data in Figure 4A and Supplementary Figure S5 are depicted as boxplots that indicate the mean ± standard deviation. In the graph, the significance levels are indicated as *p ≤ 0.05, **p ≤ 0.01, and ***p ≤ 0.001. We performed Student’s *t*-test, comparing data obtained from injected and non-injected animals (Supplementary Figure S4B).

## 3 Results

### 3.1 *In vitro* evaluation of HITI- and MITI-mediated Opto-mGluR6 knock-in (KI) in HEK293 cells

To compare the efficacies of different Cas systems to the promoter directional KI of Opto-mGluR6 downstream of the genomic ON-BPC-specific GRM6 promoter (Siegert et al., 2012), we tested two different Cas9 nucleases (SpCas9 and SaCas9) to promote HITI and two different Cpf1 nucleases (LbCpf1 and AsCpf1) to promote MITI. We first identified four target sequences within the human GRM6 gene for SpCas9, six for SaCas9, and six (three with a negative and three with a positive direction) each for LbCpf1 and AsCpf1 (Supplementary Figure S1A; Supplementary Table S1). We note that the sgRNAs are the same for LbCpf1 and AsCpf1 as they recognize the same PAM sequence essential for DNA cleavage by the nuclease. HEK293 cells were transfected with plasmids encoding a specific sgRNA driven by the U6 promoter, the respective nuclease, and the fluorescent marker eGFP (Figures 1A, B “Cas vector”). Successful Cas-mediated insertions and deletions (INDELs) were analyzed using TIDE (Supplementary Figure S1B). SpCas9—with hSpCas9-1 generating 73.8% ± 4.1% INDELs—outperformed SaCas9, with hSaCas9-2 producing 22.8% ± 0.6% INDELs. LbCpf1 was less efficient: hLbCpf1-2 produced 13.0 %± 2.1% (negative orientation) and hLbCpf1-6 22.2% ± 10.1% (positive orientation) of INDELs but still outperformed AsCpf1, which was inefficient and was therefore not further investigated.

We next set up a cytofluorimetric screening assay on murine N2a cells to evaluate the abilities of SpCas9, SaCas9, and LbCpf1 to promote HITI or MITI, respectively. We chose N2a cells since they do not possess the selected human sgRNA target sequences in their genome. For each nuclease, we transfected N2a cells with three plasmids (Figure 1A for HITI and 1B for MITI): (I) the Cas vector as used before in HEK293 cells, (II) an acceptor vector with the genomic human target sequence directionally placed between a CMV ubiquitous promoter and a bovine growth hormone poly-adenylation signal (BGHpA) preceded by a woodchuck hepatitis virus posttranscriptional regulatory element (WPRE), and (III) a donor vector containing the red fluorescent marker TurboFP635 5′ and 3′ flanked with the reverse human genomic target sequences. In the HITI strategy, Cas9 cuts the genomic target sites in both the acceptor and donor vectors. In the case of correct recombination, the target sites will be destroyed; in the case of inverted or no integration, the target sites will be formed again, thus favoring a second round of cutting which would enrich donor DNA (TurboFP635) insertions at the correct target location (Figure 1A). LbCpf1 releases sticky ends and therefore requires two neighboring genomic DNA target sites (Figure 1B). In the MITI system, only a directional DNA insertion will destroy the target sites, while a lack of recombination would reconstitute the target sites, thus favoring another round of cutting. For MITI, we modified the sgRNA sequences flanking the donor cassette to obtain complementary sticky ends after cleavage of the donor cassette and the genomic target site by the nuclease. For MITI, therefore, not only one but four sgRNAs are employed (Supplementary Table S1) and can be expressed from one single hU6 promoter since LbCpf1 can self-process arrays of sgRNAs (Zetsche et al., 2017).

As a direct indicator of recombination efficiency, we determined the ratio of TurboFP635-positive cells (where recombination took place) and eGFP-positive cells (transfected cells) by cytofluorimetric analysis (Figure 1C). SpCas9 performed best with 52.5% ± 3.1% of recombination, followed by LbCpf1 (27.1% ± 4.6%) and SaCas9 (3.2% ± 1.4%), which was not different from scrambled controls lacking the nuclease target sequence (≤4.7 ± 1.5%). The low HITI efficacy of SaCas9 was confirmed by testing another sgRNA target (data not shown), and we consequently did not proceed further with SaCas9.

In summary, all nucleases produced INDELs, but only SpCas9 and LbCpf1 were efficient at mediating HITI and MITI, respectively, and were therefore pursued further.

### 3.2 Design and evaluation of murine *in vivo* vectors

For *in vivo* HITI and MITI in the murine retina, we identified five target sequences for SpCas9 and eight target sequences for LbCpf1 (three with negative orientation and five with positive orientation) in the murine *Grm6* gene (Supplementary Figure S2A; Supplementary Table S2). INDEL formation was evaluated on murine N2a cells (Supplementary Figure S2B). All SpCas9 sgRNAs showed high INDEL formation ranging from 73.1% to 85.8%, with SpCas9 again outperforming LbCpf1. We used mSpCas9-5 (77.7% ± 11.5%) in the 5′ UTR of the *Grm6* gene (directly before the ATG start codon) and mLbCpf1-4 (negative orientation; 28.6% ± 7.5%) and mLbCpf1-8 (positive orientation; 62.3% ± 0%), both located in intron 2 of Grm6, for vector design.

The three expression cassettes used for MITI or HITI *in vivo* are the Cas nuclease, the sgRNAs, and the Opto-mGluR6 donor sequence. Together, they are too big to fit into a single AAV vector, which has a DNA packaging size limit of 4.8 kb (Hermonat et al., 1997). We therefore opted for a dual AAV vector system, where a first vector expresses the nuclease (SpCas9 or LbCpf1) and a second carries the sgRNA and Opto-mGluR6 donor cassette. As the available ON-BPC-specific promoters (Hulliger et al., 2020) are too big to fit between the AAV ITRs together with the nuclease, we set the nucleases under the control of a short CMV promoter (Figure 2A). The use of the ubiquitous CMV promoter was feasible since ON-BPC-specific expression of Opto-mGluR6 should be given by targeted KI into the *Grm6* gene, which is (within the retina) active exclusively in ON-BPCs. The donor vector in the dual AAV system includes the sgRNA(s) under the hU6 promoter and the Opto-mGluR6-IRES-eGFP optogene. To determine the donor vector configuration with the least “leak” transgene expression potentially mediated by the U6 promoter (Gao et al., 2018) or the viral ITRs (Earley et al., 2020), we designed three versions of HITI and MITI donor vectors (Figures 2A, B). In configuration I, we placed the sgRNA cassette before the donor cassette. In configurations II and III, we placed the sgRNA cassette behind the donor cassette and separated the donor cassette from the 5′ITR by either a SV40polyA sequence (configuration II) or a 180-bp spacer sequence derived from a non-coding region of the adenovirus genome (configuration III). For MITI, we removed the ATG from the Opto-mGluR6 coding region in the donor cassette but included a 5′ splice acceptor site (SAS) to promote splicing of Opto-mGluR6 behind the endogenous ATG in exon 2 of the *Grm6* gene after genomic recombination. Furthermore, we included a T2A sequence after the SAS sequence to mediate the separation of the endogenous Grm6 N-terminus from the Opto-mGluR6 protein (Figure 2B). We again modified the sgRNA target sequences in the donor vector in order to provide fully compatible sticky ends (Supplementary Table S2).

To test the three donor vector configurations *in vitro*, we designed additional acceptor vectors for HITI (Acc mHITI; Figure 2A) and MITI (Acc mMITI; Figure 2B). In Acc mHITI, we placed the mSpCas9-5 target sequence downstream of a CMV promoter; directional KI will then allow the expression of the Opto-mGluR6-eGFP cassette. To simulate the insertion of the donor cassette into an intronic region of Grm6 for MITI, we prepared a vector with a random 5′ DNA tail from the *CDH23* gene under the control of the CMV promoter, followed by the SAS and the four mLbCpf14-8 sgRNA target sequences. An ideal donor vector should possess minimal background expression of the donor cassette. To evaluate “leak” expression, we transfected HEK293 cells with all three plasmids needed for HITI/MITI and with the donor vector alone (Figures 2C, D). The donor cassette will be expressed from the CMV promoter only upon directional KI or, in case of “leak” expression, from the U6 promoter or the viral ITRs in the donor vector. eGFP expression was quantified as follows:

$$N\left(eGFP\;expressing\;cells\right)\;x\;mean\;eGFP\;intensity.$$

As can be seen in Figure 2C for HITI and Figure 2D for MITI, all three donor vector configurations led to significantly increased levels of eGFP expression when transfected together with the Cas and the acceptor vector, whereas the donor vector alone, as expected, drove significantly less eGFP expression (*p* < 0.001). This confirms the efficacy of HITI and MITI for mediating KI independently of the donor vector configuration. However, configuration I showed the highest “leak” eGFP expression for both HITI (Figure 2C) and MITI (Figure 2D), confirming that the U6 promoter can mediate some transcription of the donor cassette (Gao et al., 2018). The configuration with the significantly lowest basal expression was configuration II for HITI (*p* > 0.01) and II and III for MITI (*p* < 0.01), suggesting that the two tested ITR insulators reduced ITR-mediated transgene expression and, potentially, execute different roles in the two vectors—the reason for which, however, remains unclear.

### 3.3 Exo-AAV1 for efficient transduction of ON-Bipolar cells after intravitreal injection into the murine retina

For the delivery of the HITI and MITI systems in the retina, we opted for the AAV2 (7m8) vector (Dalkara et al., 2013) which is known to efficiently transduce ON-BPC (Ramachandran et al., 2017; Hulliger et al., 2020). For both HITI and MITI, we subretinally injected two 10-week-old C57BL/6 mice (*n* = 4 eyes) with a combination of the nuclease and donor vector (ratio = 1:1; HITI = 2 × 10^9^ vg/each vector/eye; MITI = 5 × 10^8^ vg/each vector/eye). The animals were sacrificed 6 weeks later, and histological analysis of retinal cryosections showed no eGFP expression (data not shown). We hypothesize that this lack of expression, and thus of MITI- or HITI-mediated recombination, might be due to a lack of double infection of the same cells with the two vectors. ON-BPCs are, indeed, notoriously non-permissive to AAV infection.

We therefore sought to employ Exo-AAVs, which can encapsulate several AAV virions into the same exosome promoting co-transduction of the same cell. We chose Exo-AAV1 and Exo-AAV2. Exo-AAV1 is produced using AAV serotype 1 and was shown to encapsulate up to eight AAV virions into the same exosome (Maguire et al., 2012). However, Exo-AAV1 was not yet tested in the retina. Exo-AAV2 is produced using AAV serotype 2, encapsulates two AAV virions, and was shown to efficiently transduce retinal cells (Maguire et al., 2012; Wassmer et al., 2017; Wang et al., 2021). Ideally, Exo-AAVs should be isolated from HEK293 cells co-transfected with the Cas and donor vectors. However, simultaneous transfection of the two vectors would lead to cleavage of the donor vector hampering its packaging. We therefore prepared the Cas and Donor vector containing Exo-AAVs separately but mixed them during the centrifugation step, aiming to promote fusion of the exosomes derived from the two productions.

**FIGURE 3:**
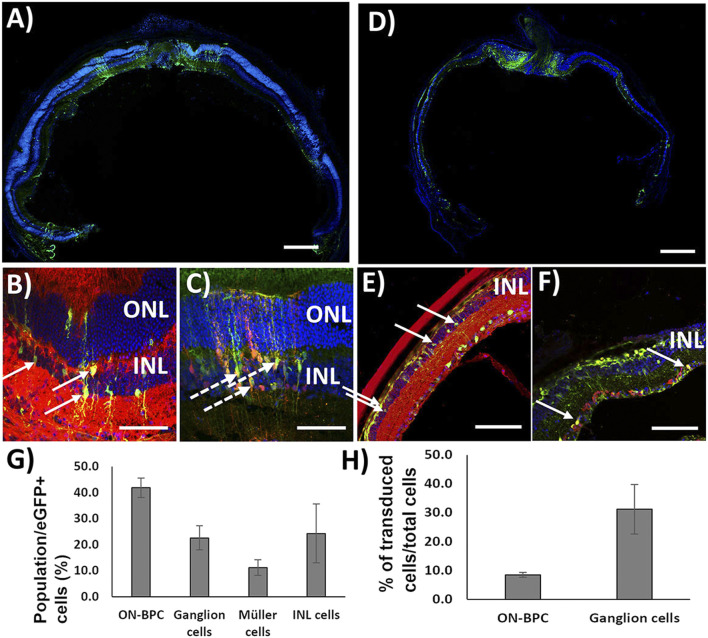
Exo-AAV1 efficiently transduces inner retinal cells upon intravitreal injection. **(A–C)** Representative confocal micrographs of retinal cryosections from C57BL/6 mice injected with Exo-AAV1 mixtures of virions expressing eGFP and TurboFP635, respectively, under the CMV ubiquitous promoter. **(A)** Tale scans of the whole retina labeled with the nuclear stain DAPI and against eGFP; **(B)** section labeled against eGFP, G0α (ON-BPC specific marker), and DAPI; **(C)** section with natural eGFP fluorescence, anti-TurboFP635, and DAPI staining. **(D–F)** Representative confocal micrographs of retinal cryosections from *rd1* mice injected with Exo-AAV1 mixtures of virions expressing eGFP and TurboFP635, respectively, under the CMV ubiquitous promoter. **(D)** Tale scans of the whole retina labeled with the nuclear stain DAPI and against eGFP; **(E)** section labeled against eGFP, G0α (ON-BPC-specific marker), and DAPI; **(F)** section labeled against eGFP, RMBP (ganglion cell-specific marker), and DAPI. **(G)** Quantification of cell-type-specific transduction. INL represents inner nuclear layer cells which are not ON-BPCs, e.g., OFF-BPCs, horizontal cells, and amacrine cells. **(H)** Quantification of the transduction efficiency in ON-BPCs and RGCs. ONL, outer nuclear layer; INL, inner nuclear layer. Full arrows indicate cells double stained for eGFP and Gαo **(B,E)** and eGFP and RBPMS **(F)**. Dashed arrows indicate cells double stained for eGFP and TurboFP635. Scale bar 200 μm **(A, D)**, 50 μm **(B, C)**, and 10 μm **(E, F)**.

First, to test the co-transduction abilities of Exo-AAV1 and Exo-AAV2, we prepared both using eGFP driven by the CMV promoter in the first AAV packaging and TurboFP635 driven by the CMV promoter in the second packaging round. A measure of 2 μl of each Exo-AAV production was injected intravitreally into C57BL/6 mice (Exo-AAV1: 1.3 × 10^10^ vg/each; N = 4. Exo-AAV2: 2.5 × 10^9^ vg/each vector/eye; *n* = 2). The animals were sacrificed 4 weeks later and the retinas cryosectioned and stained for eGFP, Gαo (an ON-bipolar cell marker), and TurboFP635. EGFP and TurboFP635 co-labeling are indicative of successful co-transduction by the two Exo-AAV preparations, while additional Gαo labeling identifies ON-BPCs. Transduction with Exo-AAV1 was found throughout the retina but mainly in hot-spots 100–200 µm in diameter (Figure 3A). Interestingly, Exo-AAV1 and Exo-AAV2 showed entirely different transduction patterns: Exo-AAV1 preferentially transduced cells of the inner nuclear layer, including ON-BPCs (Figures 3B, C), while Exo-AAV2 was selective for PRs, as shown in Supplementary Figure S3. Due to its higher selectivity for ON-BPCs, we chose exo-AAV1 for injecting 5-month-old *rd1* mice, a widely used mouse model of RP (1.6 × 10^10^ GC/each vector/eye. N = 4). The mice were sacrificed 4 weeks later for immunohistochemical analysis. The spread and localization of transduction was similar to that observed in C57BL/6 retinas (Figure 3D). We analyzed the transduction pattern of exo-AAV1 in *rd1* eyes in more detail by performing immunostaining specific for ON-BPC (Gαo, Figure 3E) and ganglion cells (RBPMS, Figure 3F), and we observed that, indeed, the majority (41.9% ± 3.7%, mean ± s.e.m.) of transduced cells were ON-BPC, 22.6% ± 4.6% were ganglion cells, and 11.2% ± 3.0% were Müller glia cells, while the remaining 24.3% ± 11.3% accounted for amacrine, horizontal, and OFF-bipolar cells (Figure 3G). We also determined the efficacy of ON-BPC and ganglion cell transduction by counting the expressing cells within each cell population. Expressing eGFP were 8.6% ± 0.9% (mean ± s.e.m.) of ON-BPCs and 31.2% ± 8.6% of ganglion cells (Figure 3H).

We next investigated a potential inflammatory response to the exo-AAV1 treatment by staining injected retinas with the inflammatory markers Iba1 (microglia), GFAP (reactive Müller glia), and the CD8+ cell marker CD11b, indicative of reactive microglia (Supplementary Figure S4). Non-injected *rd1* retinas served as controls. We observed significant increases for all three markers in the injected *rd1* retinas (Iba1: *p* = 0.014; GFAP: *p* = 0.022; CD11b *p* = 0.022), indicative of an inflammatory response elicited by either exo-AAV1, the injection procedure itself, or both.

### 3.4 Improvement of visual acuity in blind *rd1* mice upon Exo-AAV1-mediated Opto-mGluR6 KI through MITI or HITI

**FIGURE 4:**
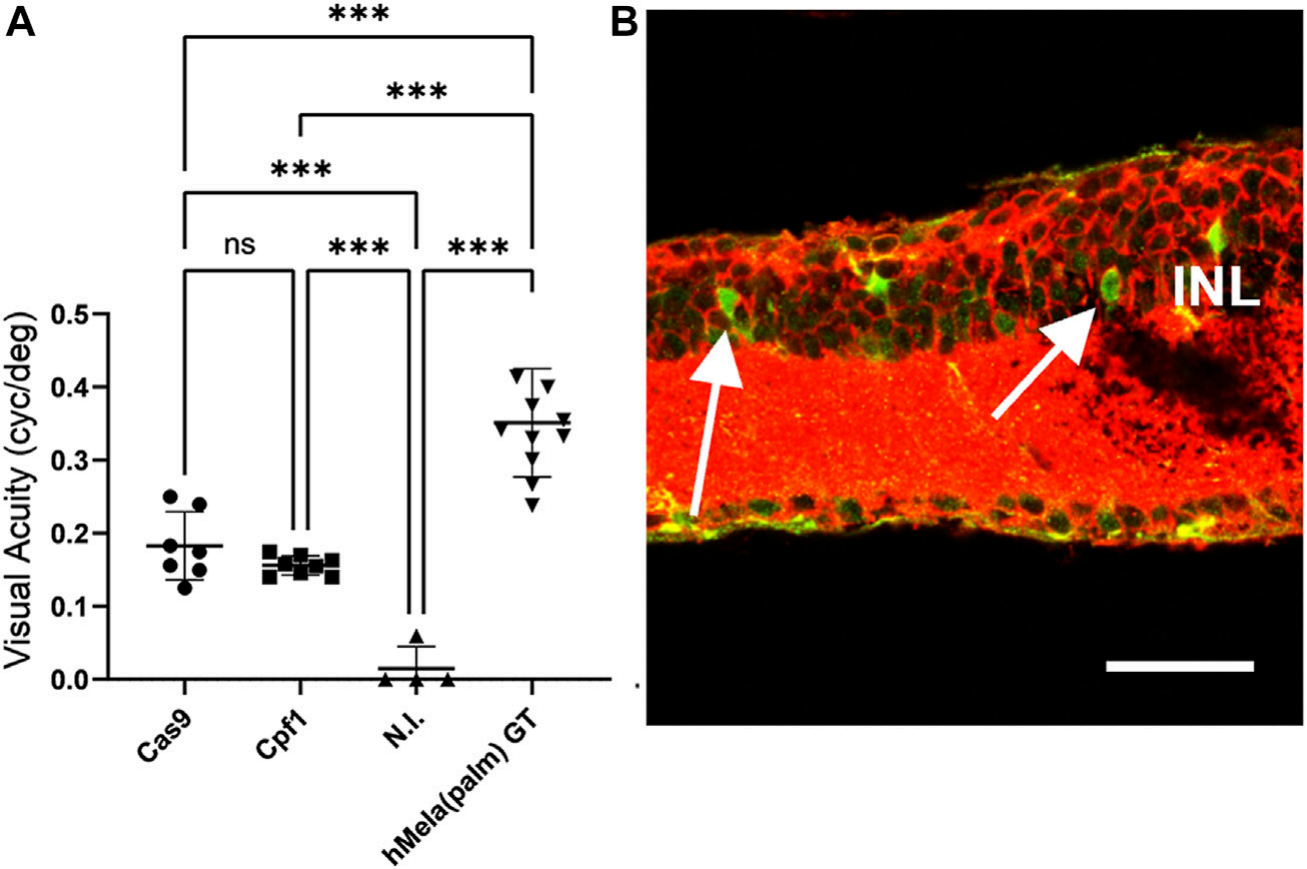
HITI- and MITI-mediated and ON-bipolar cell-targeted Opto-mGluR6 knock-in restores visual acuity in blind rd1 mice. **(A)** Visual acuity of 7-month-old rd1 mice treated with Exo-AAV1s carrying vectors for promoting Cas9-mediated HITI (HITI, *n* = 7) or Cpf1-mediated MITI (Cpf1, *n* = 8). Non-injected (N.I.) littermates (*n* = 4) and a conventional AAV Opto-mGluR6 gene therapy (Opto-mGluR6 GT, *n* = 11) served as negative and positive control, respectively. Data are depicted as boxplots indicating the mean ± standard deviation. **(B)** Representative image of a *rd1* retina transduced with the MITI Exo-AAV1 system and labeled against eGFP (transgene) and Gαo (ON-BPC marker). Double-labeled ON-BPCs (arrows) confirm successful MITI-mediated KI. INL, inner nuclear layer. Scale bar = 50 μm.

Due to the higher selectivity of Exo-AAV1 for ON-BPCs, we next evaluated Exo-AAV1-mediated HITI and MITI for *in vivo* KI of Opto-mGluR6 behind the genomic Grm6 promoter to restore vision in blind *rd1* mice. Four 5-month-old *rd1* mice lacking PRs were bilaterally and intravitreally injected with 2 μL of Exo-AAV1 containing virions, with the Cas vector and virions with the donor vector (2 × 10^10^ vg/each vector/eye) that gave the lowest background expression—for example, configuration II for HITI and configuration III for MITI. We additionally injected mice with only the donor vector [n (HITI) = 4 eyes; N (MITI) = 4 eyes] and with a combination of Cas vector and scrambled donor vector [lacking the nuclease target sites, n (HITI) = 4 eyes, N (MITI) = 6 eyes]. Six weeks post-injection, we evaluated visual acuity using the naïve optomotor kinetic reflex (OKR) test (Prusky et al., 2004). We measured a significant improvement in the visual acuity of mice injected with vectors for Cas9-mediated HITI (0.18 ± 0.02 cyc/deg, mean ± s.e.m.) and Cpf1-mediated MITI (0.16 ± 0.01 cyc/deg) compared to non-injected *rd1* controls (0.015 ± 0.03 cyc/deg; p (HITI)/p (MITI) < 0.001; Figure 4A). To estimate the efficacy of the Exo-AAV1 CRISPR systems, we also injected 11 mice with an AAV (7m8) vector carrying the Opto-mGluR6 transgene under an ON-BPC-specific GRM6 promoter (Kralik et al., 2022). The direct AAV-mediated gene supplementation therapy, as expected, restored higher visual acuities (0.32 ± 0.02 cyc/deg) than the CRISPR systems (*p* < 0.0001) due to the complexity of the latter, requiring in addition dual transduction of each ON-BPC with the Cas and the donor–vector and successful HITI or MITI to occur.

Unexpectedly, we also found in the HITI control groups ( “donor–vector only” or “scramble donor–vector plus SpCas9 vector”) a significant improvement in visual acuity (0.17 ± 0.00 cyc/deg; Supplementary Figure S5A) and a significant amount of eGFP-expressing ON-BPCs (HITI: 35.5% ± 4.5%, MITI 28.6% ± 7.5%), not significantly smaller than in the experimental Cas9 + donor–vector group (53.9% ± 8.1%, Supplementary Figure S5B). We hypothesize that transgene expression from the donor plasmid may have been mediated by the ITRs in the HITI system as the ATG start codon was kept in the Opto-mGluR6 transgene. ITR-mediated Opto-mGluR6 expression without genome integration could potentially occur in any retinal cell. Since Opto-mGluR6 is a designer protein hijacking the Gαo signaling cascade exclusive to ON-BPCs (Križaj et al., 2010; van Wyk et al., 2015), improvement of visual acuity is expected to be mediated exclusively by Opto-mGluR6 expressing ON-BPCs. In the MITI vector, the ATG was removed from the Opto-mGluR6 ORF; consequently, ITR-mediated expression is not possible. Restoration of visual acuity here is thus expected to be mediated by genomic KI of Opto-mGluR6. To confirm successful MITI-mediated Opto-mGluR6 KI into the *Grm6* gene of ON-BPCs, we performed next-generation sequencing (NGS). Five FVB/N_OptomGluR6 *rd1* mice, which expressed Turbo-FP635 exclusively in ON-BPC (van Wyk et al., 2015), were intravitreally injected with the two MITI Exo-AAV1 vectors used previously. The animals were sacrificed 5 weeks later, their retinas were dissociated, and ON-BPCs (ON-BPC+) were separated from the remainder of the retinal cells (ON-BPC-) by FACS. We performed a nested PCR around the 5′ insertion site on the isolated genomic DNA from both samples and analyzed INDEL formation. The bioinformatics analysis (Supplementary Table S3), in which we considered a region of ±50 bp around the genomic insertion point, shows 28% precise Opto-mGluR6 insertion into ON-BPC cells. It is important to note that 1) we did not observe ITR-mediated insertions, 2) that the correct allele was the most represented insertion, and 3) that, in the alleles containing INDELs, the splice acceptor site (SAS) remained intact in 97% of insertions.

We also obtained insertions in non-ON-BPCs (ON-BPC-). However, since the mGluR6 promoter is exclusively active in ON-BPCs, we consider the functional contribution of Opto-mGluR6 off-target expression in other retinal cell types a minor contributor to vision restoration. Albeit eGFP was expressed in ON-BPCs of the control groups (Supplementary Figure S5B), restoration of visual acuity in the control groups was significantly lower than that in the Cpf1 + donor treated experimental group. We therefore hypothesize that eGFP was expressed without co-expression of Opto-mGluR6 mediated by the internal ribosomal entry site (IRES) between Opto-mGluR6 and eGFP in the donor plasmid.

Overall, our results suggest that insertion of the transgene without an ATG into an intronic genomic region may be a favorable strategy for reducing “leak expression” in future *in vivo* experiments.

## 4 Discussion

The aim of this study was to knock-in the DNA sequence of the Opto-mGluR6 optogenetic protein (van Wyk et al., 2015) downstream of the genomic Grm6 promoter which is only active in ON-BPCs in order to obtain endogenously regulated and ON-BPC-specific protein expression. To promote Opto-mGluR6 genomic integration, we compared two CRISPR/Cas systems: HITI (Suzuki and Izpisua Belmonte, 2018), which releases blunt DNA ends, and MITI (Li et al., 2019), which releases sticky DNA ends. For HITI, we compared the most known and widely used SpCas9 (∼4.1 kb) with the smaller SaCas9 (∼3.1 kb), which would allow us to drive nuclease expression by ON-BPC-specific promoters (Hulliger et al., 2020), thus potentially reducing off-target expression and increasing the safety of the system. However, unlike observations by others (Pickar-Oliver et al., 2021), SaCas9 turned out to be less efficient compared to SpCas9. For MITI, we compared LbCpf1 and AsCpf1, which are similar in size (∼3.9 kb) and recognize the same PAM sequence. We compare these two nucleases solely to select the best performing; as previously reported (Ferenczi et al., 2017), this was LbCpf1. Interestingly, the *in vitro* HITI efficiency of SpCas9 was lower than the measured INDELs’ formation efficacy, while this was *vice versa* for LbCpf1—perhaps indicating the relatively higher efficiency of the MITI system. This should be tested by comparing HITI and MITI using sgRNA targets with similar INDEL values.

Our *in vitro* experiments with the HITI and MITI vectors shown in Figures 2C and D clearly indicate the endogenous ITR promotor activity suggested earlier (Earley et al., 2020), although we attempted to isolate this activity from the transgene by adding “insulators” between the ITR and the donor cassette (configurations II and III). It is interesting to note that the efficiency of the two insulators differed in the two donor cassettes used for HITI and MITI, respectively. ITR promoter activity in the HITI system was confirmed in *in vivo* experiments, where we observed leak expression of the donor vector alone (Supplementary Figure S5). In addition, we also confirmed that the U6 promoter, despite being a RNAPol (III) promoter, promotes the transcription of coding genes normally transcribed by RNAPol (II) promoters (Gao et al., 2018). Figures 2C and D show that lower “leak” transgene expression is achieved when the sgRNA expression cassette is placed after the donor cassette in the donor vector (configurations II and III).

For promoting HITI and MITI *in vivo* in ON-BPC, we used Exo-AAVs since we found dual vector transduction to be inefficient. Interestingly, Exo-AAV1 and Exo-AAV2 showed completely different patterns of transduction after intravitreal injection into the C57BL/6 mouse eye; while Exo-AAV1 efficiently targeted the inner retina—including ON-BPCs—Exo-AAV2 turned out to be specific for photoreceptors. This is surprising as the two Exo-AAVs were produced by the same HEK293 producer cell line and we would, therefore, expect a similar exosome membrane composition and thus cellular tropism. The observed difference may be explained by the different sizes of the Exo-AAVs: Exo-AAV2 contains, on average, 1.8 virions, while Exo-AAV1 contains, on average, 8.2 (Maguire et al., 2012). Due to its larger size, Exo-AAV1 may be restricted in penetrating retinal tissue and, therefore, accumulates in the inner retina after intravitreal injection. Alternatively, the AAV capsids might interact differently with the cell membrane, or there might be a different processing of the viral capsid upon cell entry. Exo-AAVs have the potential advantage over uncoated AAV particles that they escape AAV-neutralizing antibodies, which reduce the efficacy of gene therapies (György et al., 2014). The efficacy of the Exo-AAV system for CRISPR-Cas approaches could potentially be further increased by generating Exo-AAVs containing AAV particles for both the Cas and donor vectors by co-transfecting HEK293 cells with both plasmids instead of mixing the two Exo-AAV preparations during the centrifugation step. To avoid self-cutting of the vectors (and this is why we did not co-transfect), a Cas inhibitor should be used during Exo-AAV production. Notwithstanding, we show for the first time that Exo-AAV1 can be used for intravitreally mediated transduction of inner retinal cells, including ON-BPC cells that are notoriously difficult to transduce (van Wyk et al., 2017; Hulliger et al., 2020).

We employed Exo-AAV1 to evaluate HITI- and MITI-mediated Opto-mGluR6 KI *in vivo* in blind *rd1* mice. MITI and HITI significantly restored visual acuity in injected animals compared to their non-injected littermates. Although visual acuities restored by a conventional intravitreal Opto-mGluR6 supplementation gene therapy with AAV2 (7m8) (Kralik et al., 2022) were significantly higher (*p* < 0.0001, Figure 4B), the additional complexity of the CRISPR system has to be acknowledged, requiring dual transduction of ON-BPC with two different AAV capsids carrying the Cas and donor plasmid payloads, respectively, and successful subsequent HITI/MITI. Although HITI significantly restored visual acuity compared to non-injected *rd1* blind controls (Figure 4A), we also observed restoration of vision in the “donor only” and “scrambled Cas9 + donor” control groups (Supplementary Figure S5). We argue that, in the control groups injected with the donor vector, restoration is mediated by ITR-mediated Opto-mGluR6 expression from the donor plasmid. This assumption is corroborated by the fact that we found endogenous ITR promoter activity *in vitro* in the HITI system (Figures 2C, D) and that the HITI donor vector contains an ATG start codon (since targeted in the 5′UTR region of Grm6), allowing direct transcription. The MITI donor vector, however, lacks the ATG, and insertion was targeted to an intronic site (intron 2) of the *Grm6* gene, which does not allow for ITR-mediated expression. However, we also observed relatively high eGPF expression in the MITI “donor only” and “scrambled Cas9 + donor” control groups (see Supplementary Figure S5B). We believe that this eGFP expression is due to the translation of eGFP promoted by the IRES sequence between the Opto-mGluR6 and eGFP genes, without the co-translation of Opto-mGluR6 mediating the restoration of vision. For further *in vivo* vector improvement, we therefore suggest an intronic insertion for the HITI system as well. This would not only reduce leak expression of the optogene but would additionally increase the overall safety of the system since INDELs will only be introduced within the intron. For both the MITI and HITI systems, we suggest the use of the T2A sequence (mediating cleavage at the protein level) instead of the IRES sequence for eGFP linkage. Alternatively, the fluorophore could be removed altogether and successful KI could be quantified at the ON-BPC gDNA level.

Our NGS data clearly confirm successful Opto-mGluR6 KI behind the mGluR6 promoter by the MITI system (Supplementary Table S3). Although the majority of KI was exact, we still found INDELs at the insertion site, highlighting the importance of targeting the KI an intronic region and adding a splice acceptor (SAS) and T2A signal 5′ of the donor cassette. Importantly, the majority of the INDELs were smaller than 10 bp and did not affect the splicing signal. Due to the overall low efficiency of the system, we could not quantify the cells where MITI occurred. Insertions in OFF-target regions and larger genomic rearrangements should also be investigated once the overall efficiency of the system has been increased. However, we do not expect expression from cells other than ON-BPCs since the Grm6 promoter is only active in ON-BPCs.

An important concern in using gene therapy is the potential activation of the immune system, which can be triggered by the injection procedure itself, by the viral vector preparation, or by the transgene product. We observed a significant upregulation of immune markers (GFAP, CD11b, and Iba1) in retinas from injected eyes compared to retinas from non-injected eyes (Supplementary Figure S4). Although much of the immune system activation may be due to the injection procedure itself, the Exo-AAV preparation probably also elicited an immune response as our preparations did not undergo further purification steps (Cheng et al., 2021). More studies should be performed to determine the cause, severity, and possible consequences of the observed inflammation.

In conclusion, we have shown that HITI and MITI are highly efficient *in vitro* and that, despite a low *in vivo* transduction efficiency, the MITI system particularly succeeded in restoring visual acuity in otherwise completely blind *rd1* mice. In addition, we find that Exo-AAV1s efficiently mediate dual transduction of ON-BPCs after intravitreal injection.

## Data Availability

The datasets presented in this study can be found in online repositories. The names of the repository/repositories and accession number(s) can be found at: https://www.ncbi.nlm.nih.gov; ON417751-ON417756. NGS: SAMN33588014, SAMN33588015, SAMN33588016.
