# Supplementary material for: CRISPR-mediated optogene expression from a cell-specific endogenous promoter in retinal ON-bipolar cells to restore vision
Source: Front Drug Deliv. 2023 Mar 27;3:934394. doi: 10.3389/fddev.2023.934394 (PMC12363278; doi:10.3389/fddev.2023.934394)
Supplement: Supplementary file 1 [file DataSheet1.PDF]

Supplementary Table 1. List of human genome targeting sgRNAs

Human sgRNA	Location in NG_008105.1 from bp 1 to 23797. 5'→3'	Sequence 5'→3'
hSp1	4817→4836	GACTTGTCCTGAACCGACAG
hSp2	4984→4503	CGCTTGTAAGAGCGAGACGG
hSp3	4825→4806	GGACAAGTCTGTGACAGATG
hSp4	5161→5180	CAGCCCGCTAGACGAGCCGA
hSa1	6882→6860	TGGGCTCACAGAGTCCTGCCCAC
hSa2	5894→5916	CCCGCGAGGGCGCCCCTTCCCGG
hSa3	6058→6080	AGCGCTCGAGTGATATTGCAACT
hSa4	5452→5430	CAGGCGCACGCCGGGCAGCAGCT
hSa5	4991→4969	TACAAGCGCCGAACCCTGTCGCC
hSa6	4802→4824	GCCGCATCTGTCACAGACTTGTC
hCpf1 (Neg)	5812→5790	ACGCTGTGTGAATTCGAGTGATT
hCpf3 (Pos)	6090→6109	GTTTGGAACCCGGGCCCTTCTTC
hCpf4 (Neg)	6165→6143	AGGCCTATCCATCCCCCATAGCA
hCpf2 (Neg)	6197→6175	AATGATCAAAAAAGAGAAAGACC
hCpf5 (Pos)	6210→6232	CAAAATGCCTTGAACTCTCCCTC
hCpf6 (Pos)	6413→6435	GCATACAAGTTTGGGGGTTTTCG
hCpf2 (Donor)	Modified (highlighted) from hCpf2	AATGATCAAAAAAGAGAAGGTCT
hCpf6 (Donor)	Modified (highlighted) from hCpf6	GCATACAAGTTTGGGGGTCGAAA

Supplementary Table 2. List of murine genome targeting sgRNAs

Mouse sgRNA	Location in NC_000077.7 from bp 50741195 to 50757035. 5'→3'	Sequence 5'→3'
mSp1	465→484	GGCTCAGGTAAGCACCACTG
mSp2	1822→1841	AAGGCTTCTTCATCTCCAGG
mSp3	440→459	AGACTCGTCCTACTAGCCAG
mSp4	410→391	TAGAGGATTTAAGGATTCTG
mSp5	393→374	CTGGGGAGGGATGAGGGTGA
mCpf1 (Neg)	399→377	AGGATTCTGGGGAGGGATGAGGG
mCpf2 (Pos)	430→452	CAGCTGTCACAGACTCGTCCTAC
mCpf3 (Pos)	582→604	CAGGGAGAATGAGAGCAATCCTC
mCpf4 (Neg)	1358→1336	ATCTGGATGATTGGGAGAGATTG
mCpf5 (Pos)	1371→1393	GTCCACAGAGGCAGATCAATTTT
mCpf6 (Neg)	1572→1550	TCACCTACAGGTGCTCACGAGAG
mCpf7 (Pos)	1591→1613	AGGGGAACTAGGCCCTTCTTTAG
mCpf8 (Pos)	1613→1635	GAAGTCCTTAGAAGTGGCCCTGA
mCpf4 (Donor)	Modified (highlighted) from mCpf4	ATCTGGATGATTGGGAGACAATC
mCpf8 (Donor)	Modified (highlighted) from mCpf8	GAAGTCCTTAGAAGTGGCTCAGG

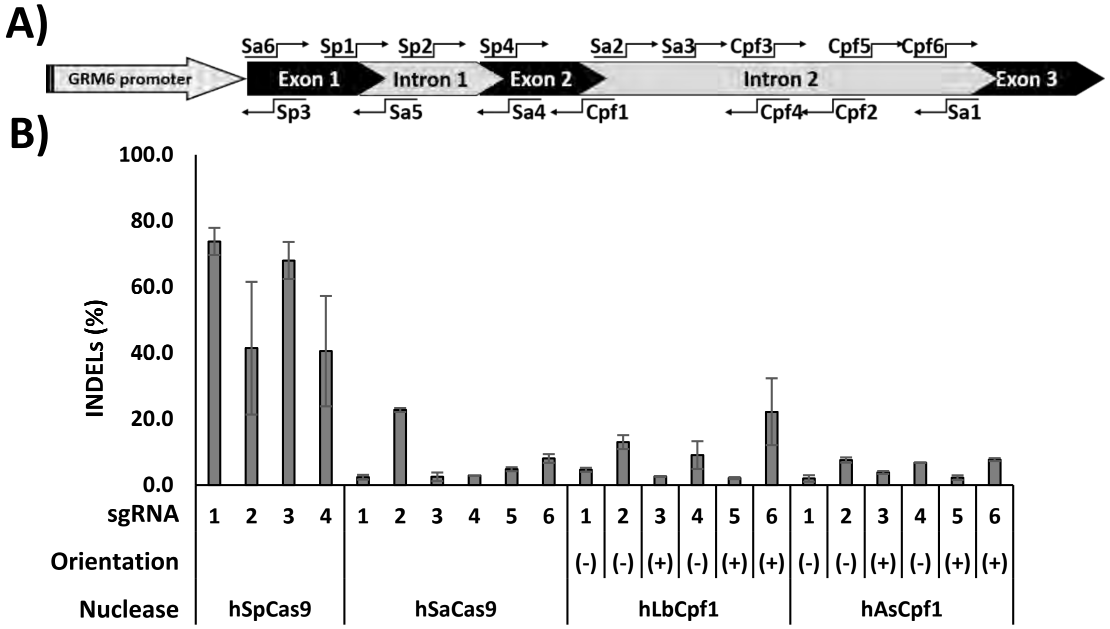

Supplementary figure S1. **Identification of sgRNAs in the human GRM6 gene.** A) Schematic representation of the human GRM6 gene and the locations of selected sgRNAs. B) INDELs quantification in HEK293 cell genomic DNA samples treated with the indicated sgRNAs and the respective nucleases. (+) and (-) indicate the orientation of the sgRNA. Results are presented as mean ± sem. N = 2.

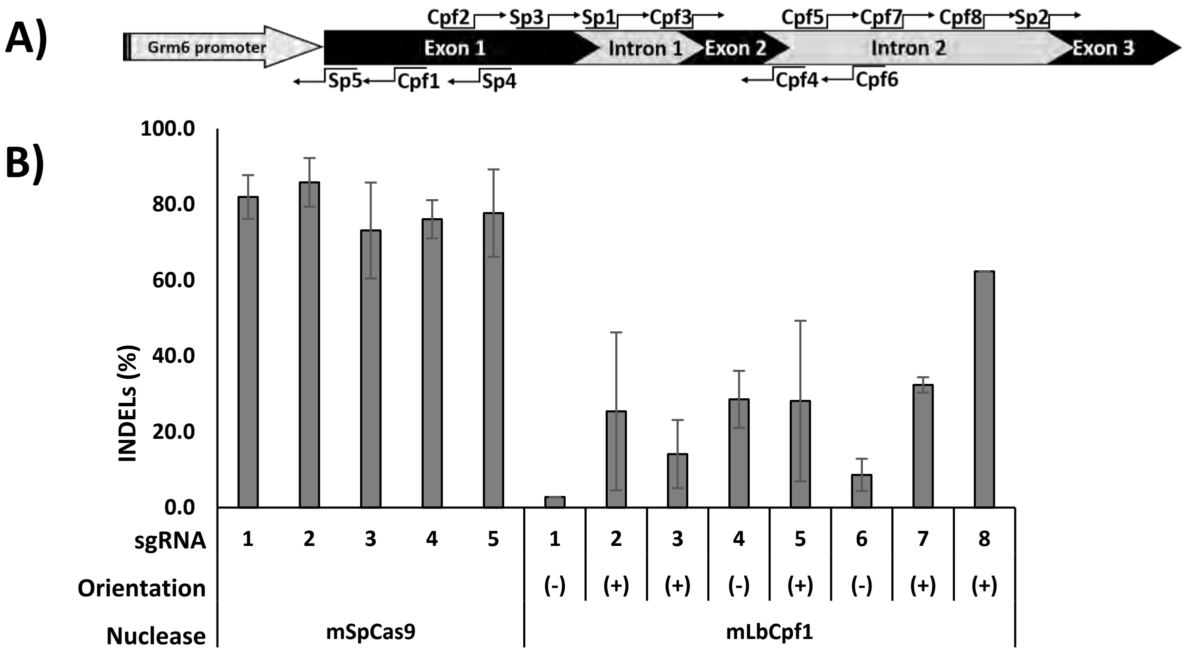

Supplementary figure S2. Identification of sgRNAs in the murine Grm6 gene. A) Schematic representation of the murine Grm6 gene and the locations of selected sgRNAs. B) INDELs quantification in N2a cell genomic DNA samples treated with the indicated sgRNAs and the respective nucleases. (+) and (-) indicate the orientation of the sgRNA. Results are presented as mean ± sem. N= 2.

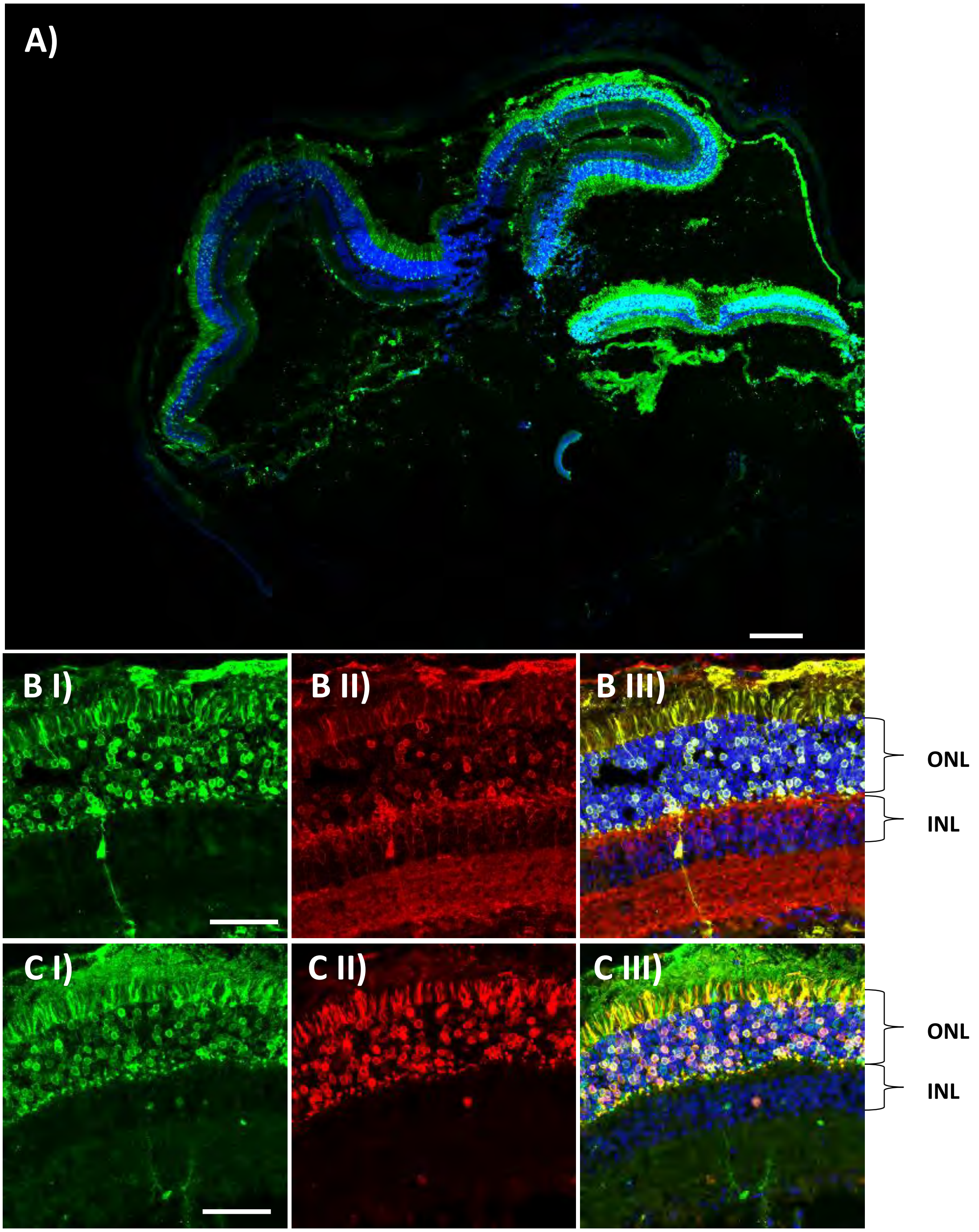

Supplementary figure S3. Exo-AAV2 efficiently and specifically transduces photoreceptors in the C57BL/6 retina. Representative examples of confocal images of retinal cryosections from C57BL/6 mice 1 months following intravitreal injection of Exo-AAV2 carrying virions expressing eGFP and TurboFP635, respectively, under the CMV ubiquitous promoter. A) Whole retinal section stained with the nuclear DAPI label and against eGFP to indicate the area of transduction. B) anti-eGFP (B I), anti-Gαo (B II, ON-BPC label), and their merge with DAPI staining (B III). C) eGFP natural fluorescence (C I), anti-TurboFP635 staining (C II), and their merge in addition to DAPI staining (C III). ONL: outer nuclear layer. INL: inner nuclear layer. Scale bar in A= 200µm, in B and C= 50µm.

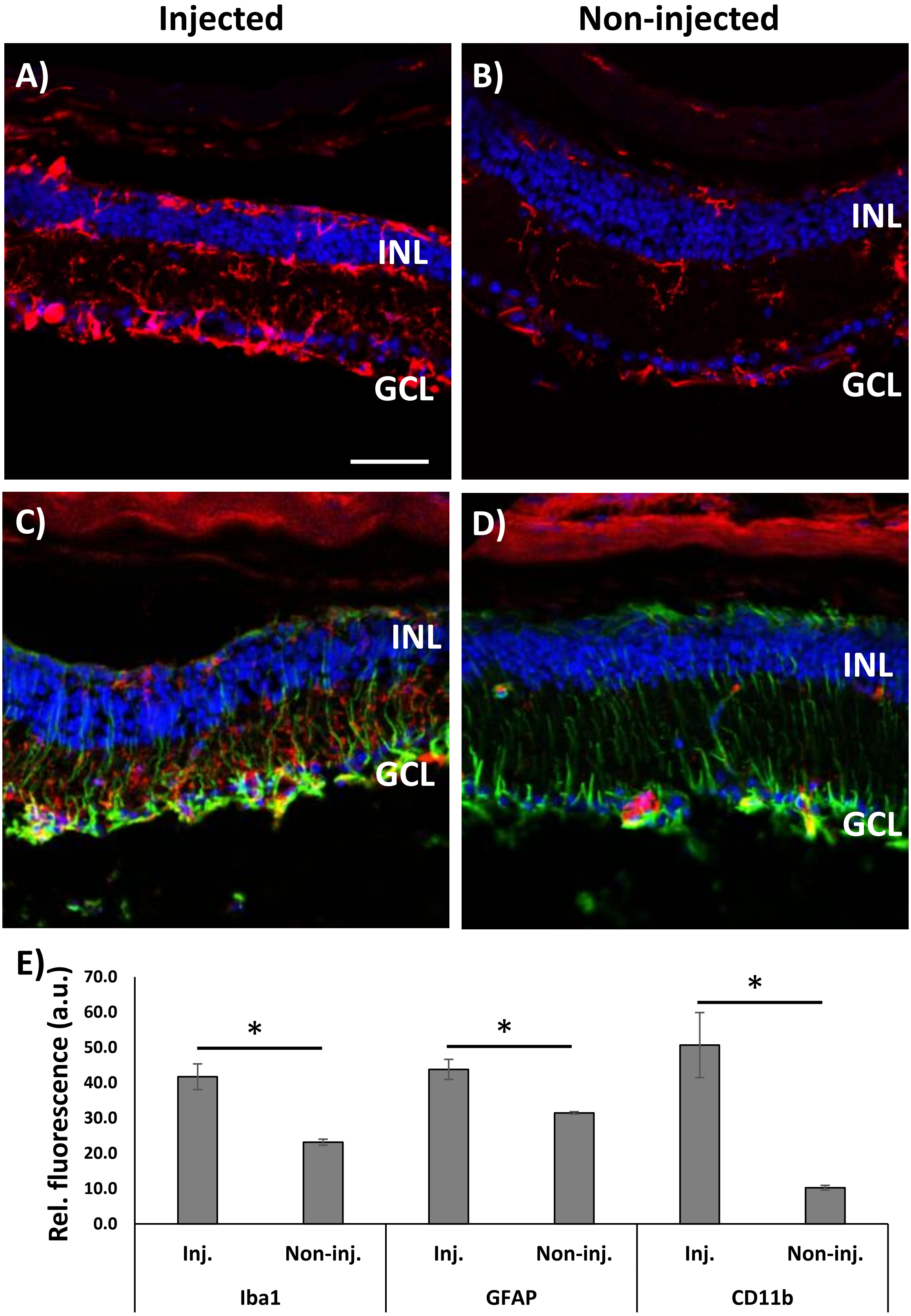

Supplementary figure S4. Exo-AAV intravitreal injection in *rd1* mice triggers immune markers. Representative examples of confocal images of retinal cryosections from 6 months-old *rd1* mice injected intravitreally (A and C) or non-injected (B and D) with Exo-AAV1s expressing eGFP and TurboFP635 under the CMV ubiquitous promoter. A,B) sections labeled against Iba1 (red, microglia marker) and DAPI. C,D) sections labeled against GFAP (green; astrocyte marker), CD11b (red; CD8+ activated microglia) and DAPI. E) Quantification of the indicated marker expression (injected eyes n=4, controls n= 2). Scale bar= 100µm, a.u. arbitrary unit, INL inner nuclear layer, GCL ganglion cell layer.

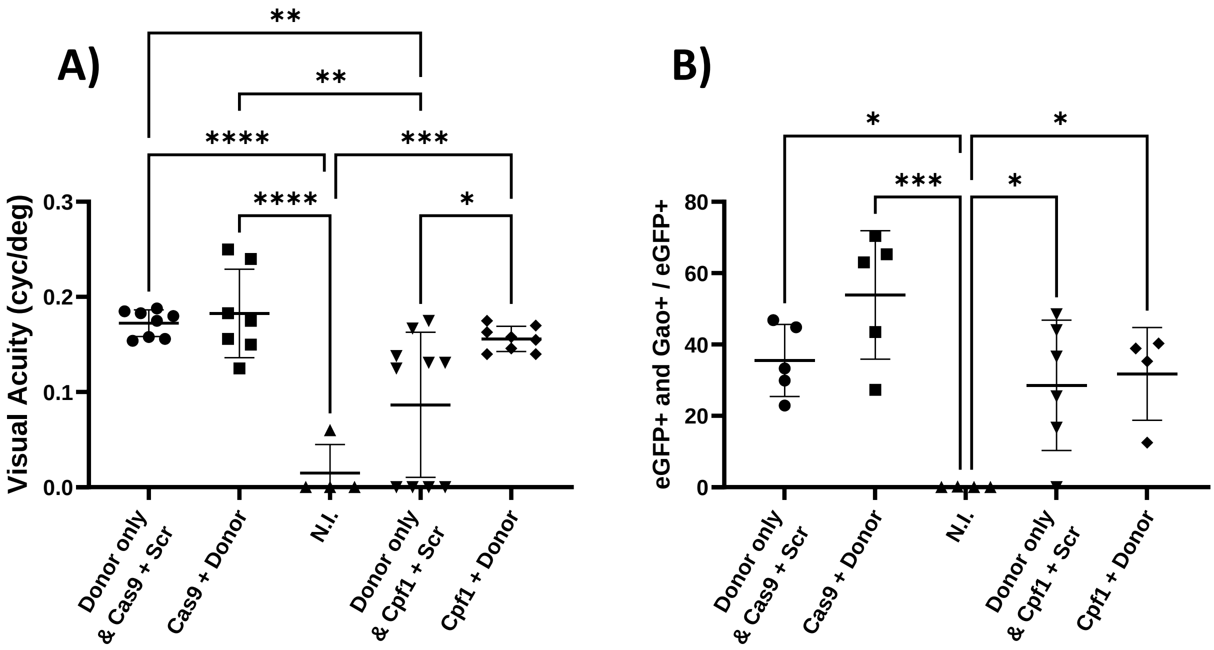

Supplementary figure S5. OFF-target expression of HITI and MITI donor vectors. A) Visual acuity determined by OKR in 7 months old *rd1* mice injected with the full set of HITI (spCas9+donor) or MITI (LbCpf1+Donor) vectors, the donor vector alone or in combination with scrambled Cas9 or not injected (N.I.). B) Quantification of ON-BPC specific eGFP expression (retinas were immunolabeled for eGFP (indicative of transgene) and Gαo (ON-BPC specific label) for quantitative counting). Results are presented as boxplots indicating the mean ± standard deviation. The number of analyzed retinas are depicted as individual data points.

Supplementary Table 3. INDELs analysis of the 5' insertion point.

Allele	ON-BPC+	ON-BPC-	N2A cells	Fasta sequence
**100M**	27,58%	54,58%	41,83%	CAGGTGGTCGATACCTTTACTAACTTCTGGAAGAATCTGTTTCAATCAAA GATAGGCACCTATTGGTCTTACTGACATCCACTTTGCCTTTCTCTCCACA
**42M1I58M**	21,52%	0,04%	0,39%	CAGGTGGTCGATACCTTTACTAACTTCTGGAAGAATCTGTTT**[C]** CAATCAAAGATAGGCACCTATTGGTCTTACTGACATCCACTTTGCCTTTCTCTCCACA
**43M1D56M**	12,79%	20,21%	0,00%	CAGGTGGTCGATACCTTTACTAACTTCTGGAAGAATCTGTTTC-**[1Del]** ACCAAAGATAGGCACCTATTGGTCTTACTGACATCCACTTTGCCTTTCTCTCCACA
**43M1D56M**	9,01%	0,03%	2,72%	CAGGTGGTCGATACCTTTACTAACTTCTGGAAGAATCTGTTTC-**[1Del]** ATCAAAGATAGGCACCTATTGGTCTTACTGACATCCACTTTGCCTTTCTCTCCACA
**39M11D50M**	6,22%	0,00%	0,00%	CAGGTGGTCGATACCTTTACTAACTTCTGGAAGAATCTG-----------**[11Del]** GATAGGCACCTATTGGTCTTACTGACATCCACTTTGCCTTTCTCTCCACA
**46M4D50M**	5,83%	0,00%	0,49%	CAGGTGGTCGATACCTTTACTAACTTCTGGAAGAATCTGTTTCAAT----**[4Del]** GATAGGCACCTATTGGTCTTACTGACATCCACTTTGCCTTTCTCTCCACA
**41M4D55M**	5,72%	0,00%	4,79%	CAGGTGGTCGATACCTTTACTAACTTCTGGAAGAATCTGTT----**[4Del]** TCAAAGATAGGCACCTATTGGTCTTACTGACATCCACTTTGCCTTTCTCTCCACA
**39M1D60M**	3,44%	7,34%	0,99%	CAGGTGGTCGATACCTTTACTAACTTCTGGAAGAATCTG-**[1Del]** TTCAATCAAAGATAGGCACCTATTGGTCTTACTGACATCCACTTTGCCTTTCTCTCCACA
**35M31D34M** *	3,42%	0,01%	0,77%	CAGGTGGTCGATACCTTTACTAACTTCTGGAAGAA-------------------------------**[31Del]** TCTTACTGACATCCACTTTGCCTTTCTCTCCACA
**46M1I54M**	3,13%	0,00%	3,66%	CAGGTGGTCGATACCTTTACTAACTTCTGGAAGAATCTGTTTCAAT**[C]** CAAAGATAGGCACCTATTGGTCTTACTGACATCCACTTTGCCTTTCTCTCCACA
**43M5D52M**	0,00%	7,59%	3,17%	CAGGTGGTCGATACCTTTACTAACTTCTGGAAGAATCTGTTTC-----**[5Del]** AAGATAGGCACCTATTGGTCTTACTGACATCCACTTTGCCTTTCTCTCCACA
**39M2D3M42D14M** *	0,00%	8,91%	0,95%	CAGGTGGTCGATACCTTTACTAACTTCTGGAAGAATCTG--**[2Del]**TCA--**[42Del]** CCTTTCTCTCCACA
**42M5I58M**	0,01%	0,00%	2,83%	CAGGTGGTCGATACCTTTACTAACTTCTGGAAGAATCTGTTT**[CAATC]** CAATCAAAATAGGCACCTATTGGTCTTACTGACATCCACTTTGCCTTTCTCTCCACA
**8M48D44M** *	0,01%	0,01%	2,72%	CAGGTGGT--**[48Del]** CACCTATTGGTCTTACTGACATCCACTTTGCCTTTCTCTCCACA
**41M4I59M**	0,00%	0,00%	2,51%	CAGGTGGTCGATACCTTTACTAACTTCTGGAAGAATCTGTT**[TCAA]** TCAATCAAAGATAGGCACCTATTGGTCTTACTGACATCCACTTTGCCTTTCTCTCCACA
**46M1I54M**	0,00%	0,00%	2,49%	CAGGTGGTCGATACCTTTACTAACTTCTGGAAGAATCTGTTTCAAT**[G]** CAAAGATAGGCACCTATTGGTCTTACTGACATCCACTTTGCCTTTCTCTCCACA
**42M2D56M**	0,00%	0,00%	2,47%	CAGGTGGTCGATACCTTTACTAACTTCTGGAAGAATCTGTTT-**[1Del]** ATCAAAGATAGGCACCTATTGGTCTTACTGACATCCACTTTGCCTTTCTCTCCACA
**42M4I58M**	0,01%	0,00%	2,17%	CAGGTGGTCGATACCTTTACTAACTTCTGGAAGAATCTGTTT**[CACA]** CAATCAAAGATAGGCACCTATTGGTCTTACTGACATCCACTTTGCCTTTCTCTCCACA
**37M9D54M**	0,00%	0,00%	2,06%	CAGGTGGTCGATACCTTTACTAACTTCTGGAAGAATC---------**[9Del]** CAAAGATAGGCACCTATTGGTCTTACTGACATCCACTTTGCCTTTCTCTCCACA
**33M20D47M** *	0,00%	0,00%	2,05%	CAGGTGGTCGATACCTTTACTAACTTCTGGAAG--------------------**[20Del]** AGGCACCTATTGGTCTTACTGACATCCACTTTGCCTTTCTCTCCACA
**Total**	98,69%	98,72%	79,06%	

Legend: In the allele column: "n"M= number of nucleotides matching with the expected sequence; "n"I= number of inserted nucleotides compared with the expected sequence; "n"D= number of deleted nucleotides compared with the expected sequence. In the FASTA sequence column the number of deleted and inserted nucleotides are indicated between brackets. The dashed line indicates the beginning of the splicing acceptor signal. The * indicates the alleles with a corrupted splicing acceptor signal. *ON-BPC+* ON-bipolar cells, *ON-BPC-* remaining retinal cells.
